# Muscles provide protection during microbial infection by activating innate immune response pathways in *Drosophila* and zebrafish

**DOI:** 10.1242/dmm.022665

**Published:** 2016-06-01

**Authors:** Arunita Chatterjee, Debasish Roy, Esha Patnaik, Upendra Nongthomba

**Affiliations:** Department of Molecular Reproduction, Development and Genetics, Indian Institute of Science, Bangalore 560012, India

**Keywords:** Muscle, *Drosophila*, Zebrafish, Infection, Immunity, Anti-microbial peptides

## Abstract

Muscle contraction brings about movement and locomotion in animals. However, muscles have also been implicated in several atypical physiological processes including immune response. The role of muscles in immunity and the mechanism involved has not yet been deciphered. In this paper, using *Drosophila* indirect flight muscles (IFMs) as a model, we show that muscles are immune-responsive tissues. Flies with defective IFMs are incapable of mounting a potent humoral immune response. Upon immune challenge, the IFMs produce anti-microbial peptides (AMPs) through the activation of canonical signaling pathways, and these IFM-synthesized AMPs are essential for survival upon infection. The trunk muscles of zebrafish, a vertebrate model system, also possess the capacity to mount an immune response against bacterial infections, thus establishing that immune responsiveness of muscles is evolutionarily conserved. Our results suggest that physiologically fit muscles might boost the innate immune response of an individual.

## Abstract (precis)

**Summary:** Using fruit fly and zebrafish models, we show that skeletal muscles are immune responsive tissues; they mount innate immune responses during bacterial infection – an evolutionarily conserved defense mechanism.

## INTRODUCTION

Host defense mechanisms against invading pathogens have evolved over time, resulting in higher vertebrates with extremely complex, multi-layered immune systems. Despite decades of research, our understanding of the immune system is not yet complete. With its powerful genetic and genomic tools and absence of typical adaptive immunity, *Drosophila melanogaster* has proved to be an excellent model for studying the intricacies of innate immunity (Brennan and Anderson, 2004; Kounatidis and Ligoxygakis, 2012; Igboin et al., 2012).

The *Drosophila* immune system is fairly well studied and is conventionally categorized into a cellular arm and a humoral arm. The cellular arm mainly comprises phagocytosis by plasmatocytes (predominant blood cells) (Lemaitre and Hoffmann, 2007; Kounatidis and Ligoxygakis, 2012). Anti-microbial peptides (AMPs), short cationic peptides that limit the growth of the invading microbes by forming pores or by other unknown mechanisms, are primarily produced by the fat body and are the hallmark of humoral immunity (Imler and Bulet, 2005; Brogden, 2005). Before systemic infection by a microbe, the boundary defense, comprising the cuticle and localized production of AMPs by the epithelium, takes care of several intrusions (Brey et al., 1993; Davis and Engstrom, 2012). Tissues endowed with this defense include the reproductive tract, tracheae, gastrointestinal tract and malpighian tubules (Tzou et al., 2000; Verma and Tapadia, 2012; Davis and Engstrom, 2012). Once a pathogen breaches these defenses, the fat body-mediated systemic AMP response in the hemocoel takes over.

Besides the fat body, *Drosophila* may also utilize other tissues for immune response. Several studies in *Drosophila* suggest that muscles affect multiple physiological processes apart from their contractile function. Although muscle-specific signaling pathways regulate the organism's oxidative stress resistance and lifespan (Tohyama and Yamaguchi, 2010; Demontis and Perrimon, 2010; Vrailas-Mortimer et al., 2011), whether muscles play a role in surviving an infection is not known. Interestingly, expression of muscle structural genes, like *actin*, *flightin*, *troponin I* (also known as *wings up A*), *troponin C* etc., are induced early on as a defense response of *Drosophila* to *Pseudomonas aeruginosa* pathogenesis (Apidianakis et al., 2005). These genes are speculated to have a role in tissue reconstruction upon trauma that acts in concert with immune responses (Apidianakis et al., 2007). However, these genes are not induced upon sterile injury where, intuitively, expression of tissue-reconstruction genes should be increased (Apidianakis et al., 2007). This suggests that muscles might have a more direct role in immunity.

To decipher the function of muscles in immunity we chose the indirect flight muscles (IFMs) of *Drosophila* as a model system because several of the muscle genes induced upon infection are specific to IFM (Apidianakis et al., 2005). Moreover, presence of several IFM-specific isoforms of many structural genes allows the manipulation of the IFMs without affecting other body muscles (Nongthomba et al., 2004, 2007). IFMs are structurally similar to vertebrate skeletal muscles and physiologically similar to cardiac muscles (Peckham et al., 1990; Moore, 2006), allowing the possibility of extrapolating the findings to higher organisms.

Here, we report that IFMs of *Drosophila* are capable of producing AMPs, and that this immune response mounted by IFMs is essential for flies to survive bacterial infection. We further establish that vertebrate skeletal muscles also respond to an immune challenge.

## RESULTS

### IFMs are required for surviving microbial infections

**Fig. 1.**
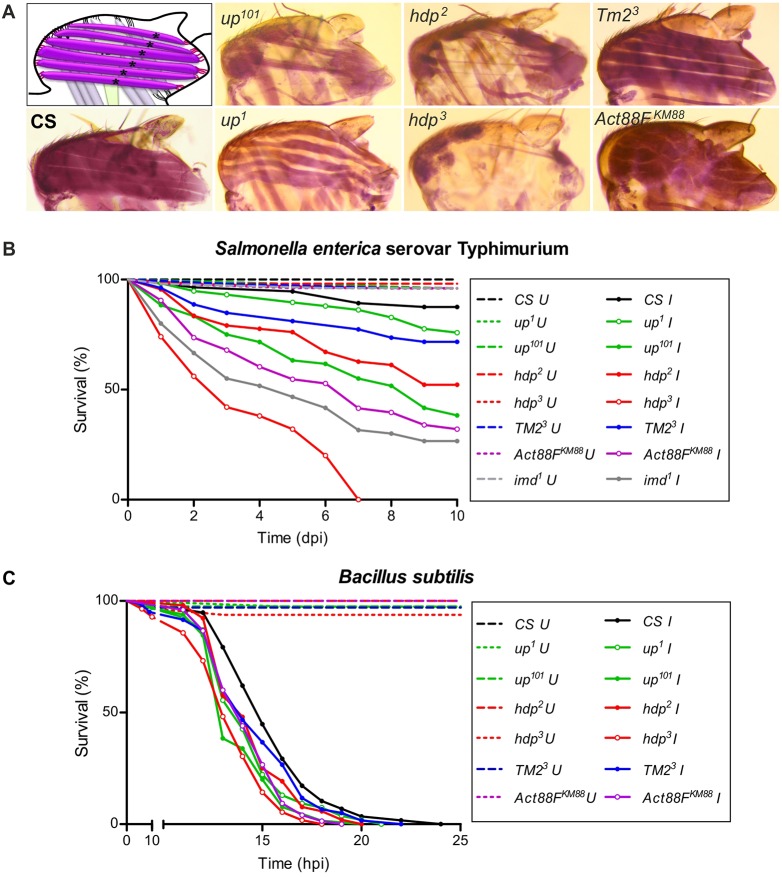
**IFM mutants are susceptible to bacterial infection.** (A) Hematoxylin-stained hemithoraces of the fly strains used in the study, alongside a representative schematic, show a spectrum of defects in the IFMs. CS: the wild-type fly strain *Canton-S*. (B,C) Survival curves of the muscle mutants post-infection with (B) *Salmonella* and (C) *Bacillus subtilis* by septic injury. A log-rank test was done for estimating the significance of the survival curves. (B) *Salmonella* infected versus uninfected: *Canton-S*, *P*=0.0094; *up^1^*, *P*=0.0034; *up^101^*, *P*<0.0001; *hdp^2^*, *P*<0.0001; *hdp^3^*, *P*<0.0001; *TM2^3^*, *P*=0.0006; *Act88F^KM88^*, *P*<0.0001. Mutant versus wild type: *up^1^*, *P*=0.1171; *up^101^*, *P*<0.0001; *hdp^2^*, *P*<0.0001; *hdp^3^*, *P*<0.0001; *TM2^3^*, *P*=0.0364; *Act88F^KM88^*, *P*<0.0001. (C) *Bacillus* infected versus uninfected: All fly strains, *P*<0.0001. Mutant versus wild type: *up^1^*, *P*=0.0139; *up^101^*, *P*=0.0002; *hdp^2^*, *P*=0.0346; *hdp^3^*, <0.0001; *TM2^3^*, *P*=0.1646; *Act88F^KM88^*, *P*=0.0009. *n*>80. Open circles, IFM-specific mutants; dashed lines, uninfected controls; U, uninfected; I, infected; dpi, days post-infection; hpi, hours post-infection.

Mutants of *Drosophila* muscle structural genes were selected for the study. Being flightless, these mutants could not be distinguished at the functional level (Fig. S1D); however, at the structural level, they showed defects of the IFMs that varied through a spectrum amongst the mutants (Fig. 1A). Most mutants showed hypercontraction (*up^1^*, *up^101^*, *wupA^hdp−2^*, *wupA^hdp−3^*) (Nongthomba et al., 2003, 2004, 2007), hypomorph for Tropomyosin *Tm2^3^* resembled the wild type in IFM morphology despite being flightless, IFM-specific actin-null *Act88F^KM88^* had a wavy appearance of the IFMs, and IFM-specific Troponin I-null *wupA^hdp−3^* had the most severely affected IFMs with no muscles (Fig. 1A).

As compared with wild-type strain *Canton-S*, all the muscle structural gene mutants (‘muscle mutants’ hereafter) showed reduced survival upon infection by septic injury, both with Gram-negative *Salmonella* infection (Fig. 1B) and Gram-positive *Bacillus* infection (Fig. 1C), or by continuous feeding with Gram-negative *Escherichia coli* (Fig. S1E). Interestingly, the susceptibility to infection was not dependent on whether the mutation was manifested throughout the body or IFM-specific. Rather, the order of susceptibility was proportional to the increasing order of defects in IFMs.

The data was evaluated statistically to assess the degree of correlation between IFM defects and susceptibility to infection. Analysis of sarcomere length and IFM area (Fig. S1A-C) allowed us to rank the mutants in terms of overall defects (Fig. S2B). Mutants were also ranked for their survival after infection (Fig. S2A). Upon correlating all the susceptibility ranks with the gross IFM defect ranks, a positive correlation coefficient of r=0.8199 (*P*=0.0003) was obtained, where the number of pairs compared was 14 (Fig. S2C,D). A statistically significant correlation between increased IFM defects and reduced survival of flies after bacterial infection suggests an important role of IFMs in surviving infection challenges.

### Optimal infection-induced AMP production requires intact IFMs

**Fig. 2.**
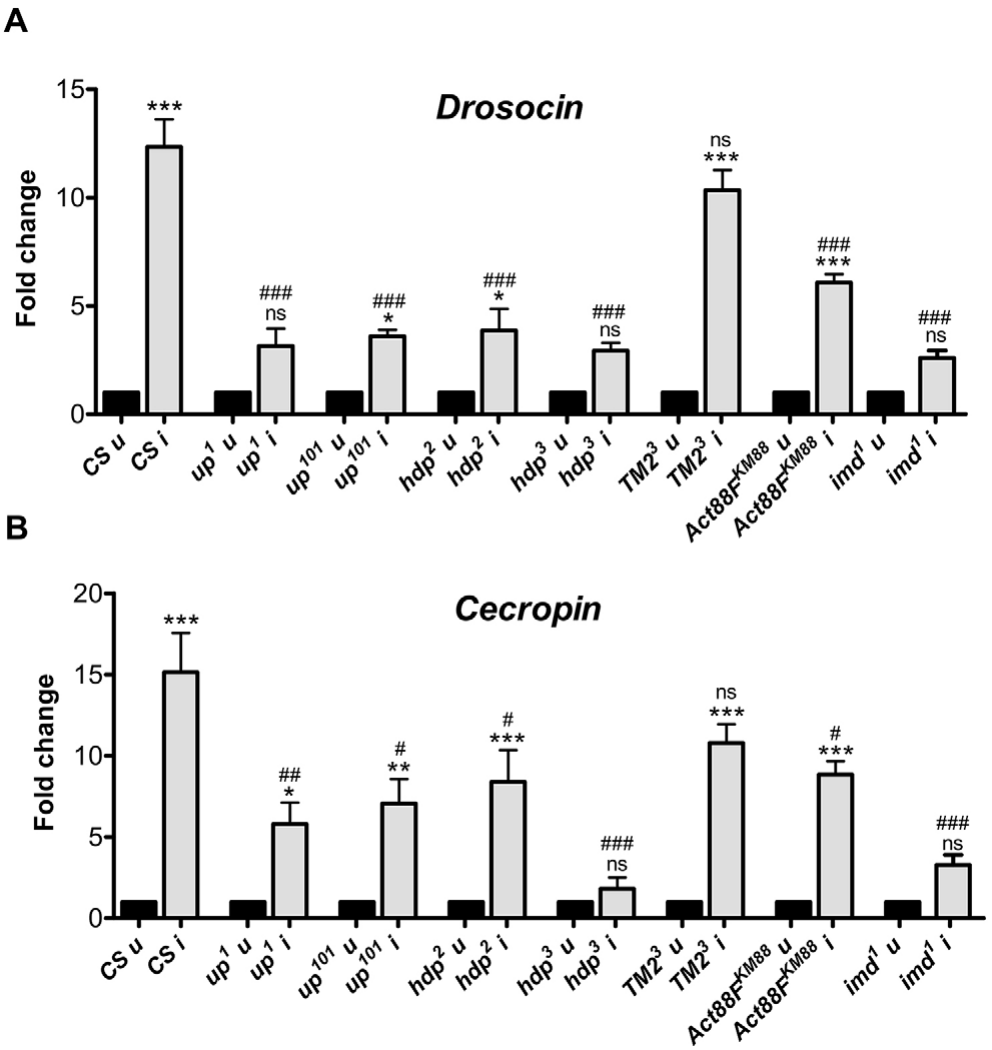
**Induction of AMPs in muscle mutants upon infection is sub-optimal.** (A,B) qRT-PCR analyses for estimation of induction of gene expression for anti-microbial peptides (A) *Drosocin* and (B) *Cecropin* upon infection. The mRNA was extracted from whole flies 6 h after infection with *Salmonella*. The values represented are mean±s.e.m. of three independent replicates. *, significant difference in expression of infected versus uninfected; #, significant difference in induction levels of infected mutant versus infected wild type. ns, *P*>0.05; */#, *P*<0.05; **/##, *P*<0.001; ***/###, *P*<0.0001. CS, the wild-type fly strain *Canton-S*; u, uninfected; i, infected.

Upon bacterial infection, the *Drosophila* innate immune system induces the expression of a specific set of AMPs directed against the infecting bacteria by primarily activating either the Toll or the immune deficiency (Imd) pathway (Lemaitre et al., 1997). Cecropin and Drosocin, two AMPs downstream of the Imd pathway, show a substantial increase in their expression six hours post-infection with *Salmonella* (Fig. 2A,B). In most muscle mutants, AMPs induction was diminished compared with wild-type flies post-infection (Fig. 2A,B); although basal expression of AMPs was comparable to that of naïve wild-type flies (Fig. S3A). *wupA^hdp−3^* flies demonstrated the weakest response to infection with no induction of either AMP. In *Tm2^3^* flies, increases in expression of both the AMPs were comparable to wild type (Fig. 2A,B), and AMP induction in the other mutants was moderate. As would be expected, reduced AMP induction correlated well with both reduced infection survival (Figs 1, 2; *n*=28, Spearman r=0.607, *P*=0.0006) and the degree of IFM defect (Fig. 2; Fig. S2; *n*=14, Spearman r=0.847, *P*=0.0001).

### IFMs produce AMPs upon infection

**Fig. 3.**
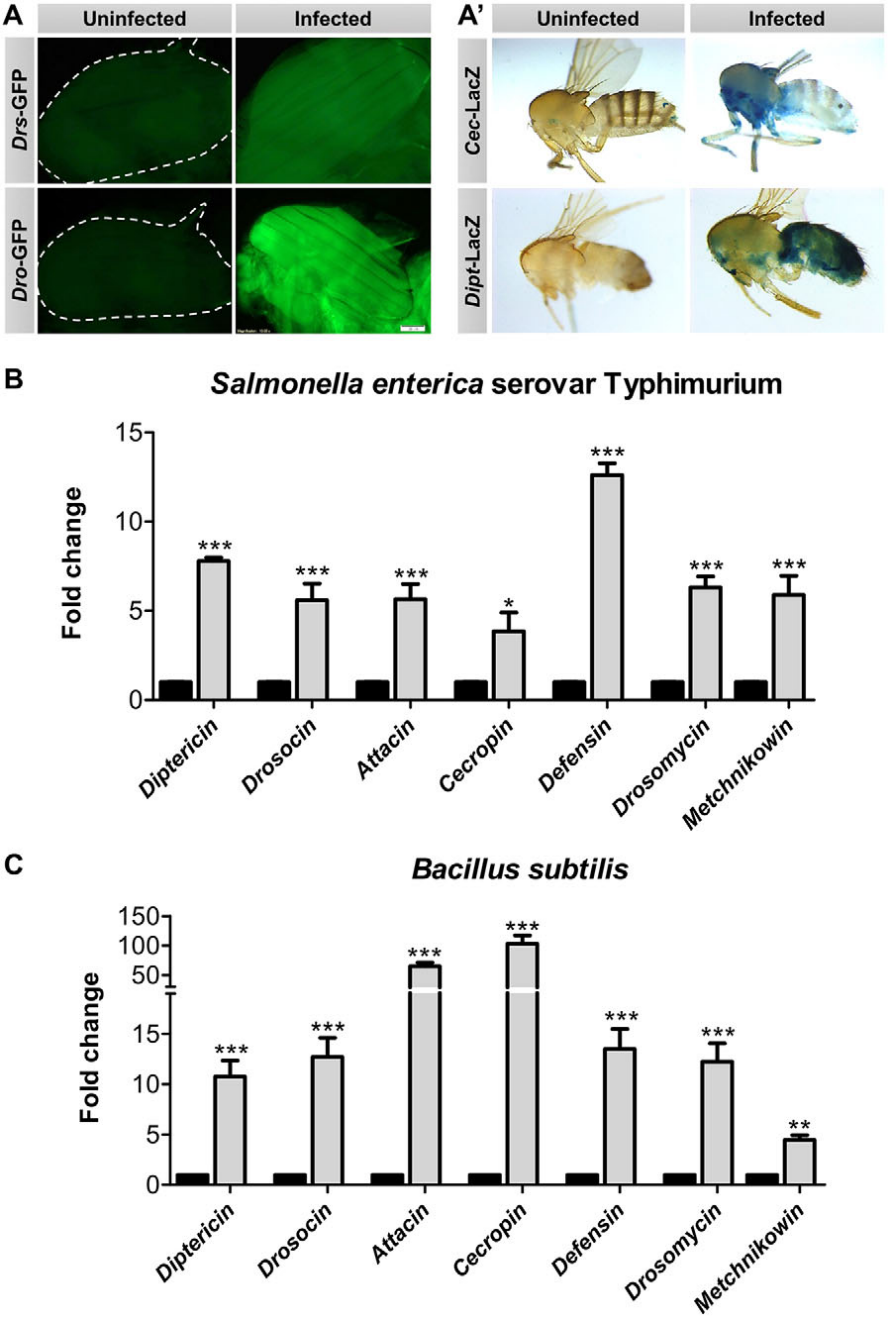
**IFMs induce AMPs upon infection.** (A,A′) Infected AMP reporter fly strains show increased expression of AMPs in their IFMs compared with naïve IFMs. *Drs*-GFP (*Drosomycin*) flies were infected with Gram-positive *Staphylococcus aureus*. *Dro*-GFP (*Drosocin*), *Dipt*-LacZ (*Diptericin*) and *Cec*-LacZ (*Cecropin*) flies were infected with Gram-negative *Enterobacter cloacae*. Flies were visualized 12 h post-infection. Scale bar: 100 µm. (B,C) Change in expression of the seven *Drosophila* AMPs in the IFMs of wild-type flies when infected with (B) *Salmonella* or (C) *Bacillus subtilis*. The mRNA was isolated from IFMs 6 hpi. Solid columns represent uninfected controls for each of the genes tested. The values represented are mean±s.e.m. of three independent replicates. *, significant difference in expression of infected versus uninfected. *P<0.05; **P<0.001; ***P<0.0001.

The loss of induction of AMPs in muscle mutants indicated that IFM might be an immunologically active tissue that produces AMPs upon infection. To test this hypothesis, we took two approaches. Firstly, flies with AMP reporter constructs were infected to visualize if any expression could be identified in IFM. Expression of the tested AMPs – Drosocin, Drosomycin, Cecropin and Diptericin – were elevated in the IFMs of infected flies, and also in other known body organs viz. fat and epithelia (Fig. 3A,A′).

Secondly, the induction of AMPs in the IFMs of infected wild-type flies was assessed six hours post-infection by quantitative reverse transcriptase PCR (qRT-PCR). All seven AMPs of *Drosophila*, viz. Attacin, Cecropin, Diptericin, Drosocin, Defensin, Metchnikowin and Drosomycin, were induced in IFMs upon *Salmonella* infection (Fig. 3B). Interestingly, the levels of AMPs responsive to Gram-positive bacteria and/or fungi (Defensin, Drosomycin and Metchnikowin) were also elevated in IFMs after a Gram-negative bacterial infection (Fig. 3B). Similar induction of all the AMPs, though at a higher level, was observed when the wild-type flies were infected with a Gram-positive bacterial species, *Bacillus subtilis* (Fig. 3C). *Bacillus subtilis* has DAP-type peptidoglycans (ligands of Gram-negative bacteria used to activate specific immune response) and thus, infection also caused induction of Gram-negative bacteria-responsive AMPs. This showed that IFMs are indeed an immune responsive tissue and can produce AMPs against both Gram-negative and Gram-positive bacteria.

### IFMs produce AMP upon infection using canonical signaling pathways

**Fig. 4.**
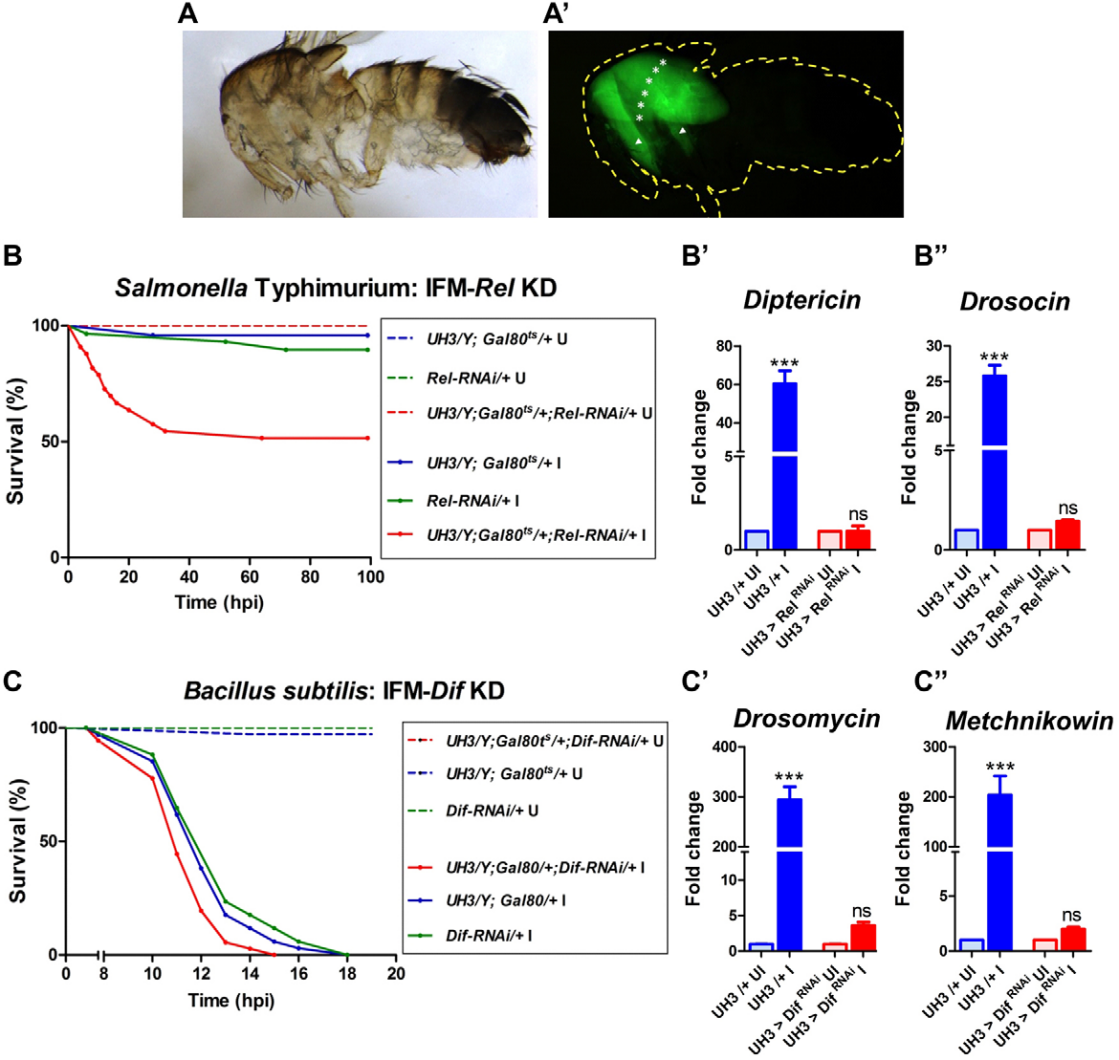
**IFM-mediated immune response is essential for survival upon bacterial infection.** (A,A′) Expression of *UH3-Gal4* is restricted to IFMs in 2-day-old adults. Pattern of expression was visualized with UAS-GFP as a reporter. Yellow dotted line represents the body outline of the fly. White asterisks represent the dorsal longitudinal muscles and arrowheads indicate the dorso-ventral muscles, which together constitute the IFM. Also see Fig. S4. (B,C) Survival of male flies (*n*>50) infected with (B) *Salmonella* and (C) *Bacillus subtilis*. A log-rank test was done for estimating the significance of the survival curves. (B) *Salmonella* infected versus uninfected: *UH3-Gal4*/+ (*Gal4* control), *P*=0.2207; *Rel-*RNAi/+ (UAS control), *P*=0.0640; *UH3*>*Rel*-RNAi (test), *P*<0.0001. Test versus infected *Gal4* control, *P*=0.0003. Test versus infected UAS control, *P*=0.0009. (C) *Bacillus* infected versus uninfected: All fly strains, *P*<0.0001. Test (*UH3*>*Dif*-RNAi) versus infected *Gal4* control (*UH3-Gal4*/+), *P*=0.0381. Test versus infected UAS control (*Dif-*RNAi/+), * P*=0.0046. Solid lines, infected flies; dashed lines, uninfected controls; KD, knockdown; U, uninfected; I, infected, (B′,B″,C′,C″) AMP gene induction in the IFMs upon infection as tested by qRT-PCR of tissues isolated at 6 hpi. Induction of (B′) *Diptericin* and (B″) *Drosocin* was entirely lost in flies with altered IFM-Imd signaling. Similarly increased expression of (C′) *Drosomycin* and (C″) *Metchnikowin* was entirely lost in flies with altered IFM-Toll signaling*.* The values represented are mean±s.e.m. of three replicates. *, significant difference in expression of infected versus uninfected. ns, *P*>0.05; ***P<0.0001.

To test if the AMP-production response of IFMs upon infection is via canonical signaling pathways (Toll and Imd), we took a genetic approach. The transcription factors downstream of the Toll and Imd pathways, Dorsal-related immunity factor (Dif) and Relish (Rel), respectively, were knocked down individually under spatio-temporal control (i.e. only in adult IFMs) using the *Gal4-*UAS system (Brand and Perrimon, 1993). We used *UH3-Gal4*, whose expression is restricted to IFMs in the adult flies (Fig. 4A,A′; Fig. S4) (Singh et al., 2014). The knockdown was specifically done in the adults as the Toll and Imd pathways are important during development. We predicted that the IFMs of infected test flies (with either *Dif* or *Rel* knocked down in IFMs) will fail to induce the effector molecules or AMPs, though AMPs from other immune organs will be induced unhindered, and thus survival post-infection might be reduced as compared with controls. Alternatively, if IFMs activate Toll- or Imd-independent pathways for inducing AMPs, there would be no effect of *Dif* or *Rel* knockdown on survival post-infection.

*Dif* knockdown in IFMs significantly reduced the survival of flies upon infection with *Bacillus subtilis* (Fig. 4C). In agreement with the reduced survival, IFMs of these flies were unable to induce the Toll pathway-specific AMP genes, *Drosomycin* and *Metchnikowin*, post infection (Fig. 4C′,C″). Elevation in transcript levels of *Defensin*, an AMP downstream of both the Toll and Imd pathways, was also moderate (Fig. S5A). Similarly, *Rel* knockdown in IFM drastically reduced the survival of flies upon infection with *Salmonella* (Fig. 4B). Confirming the survival data, the IFMs of the flies with *Rel* knockdown were unable to induce AMP genes specific to the Imd pathway (*Diptericin* and *Drosocin*) (Fig. 4B′,B″). *Cecropin*, encoding an AMP downstream of both the Toll and Imd pathways, was induced, albeit to a reduced level (Fig. S5B).

Thus, AMPs are produced by IFMs through activating canonical immune signaling pathways and these IFM-produced AMPs are essential for the survival of flies upon infection.

### Induction of TLR/NFκB signaling in muscles upon infection is conserved in vertebrates

IFMs of *Drosophila* are structurally similar to vertebrate skeletal muscles. This led to the possibility that active immune signaling networks might be conserved in vertebrate skeletal muscles. To test the same, adult *Danio rerio* (zebrafish) trunk muscles were taken as a representation of vertebrate skeletal muscles. Unlike *Drosophila*, NFκB (homologous to Toll signaling) and TNFα (homologous to Imd signaling) signaling in vertebrates leads to expression of cytokines and not AMPs (Pahl, 1999; Zhou et al., 2003). Therefore, we assessed the induction of cytokines (both pro- and anti-inflammatory) in trunk muscles of zebrafish with *Salmonella* (Fig. S7).

**Fig. 5.**
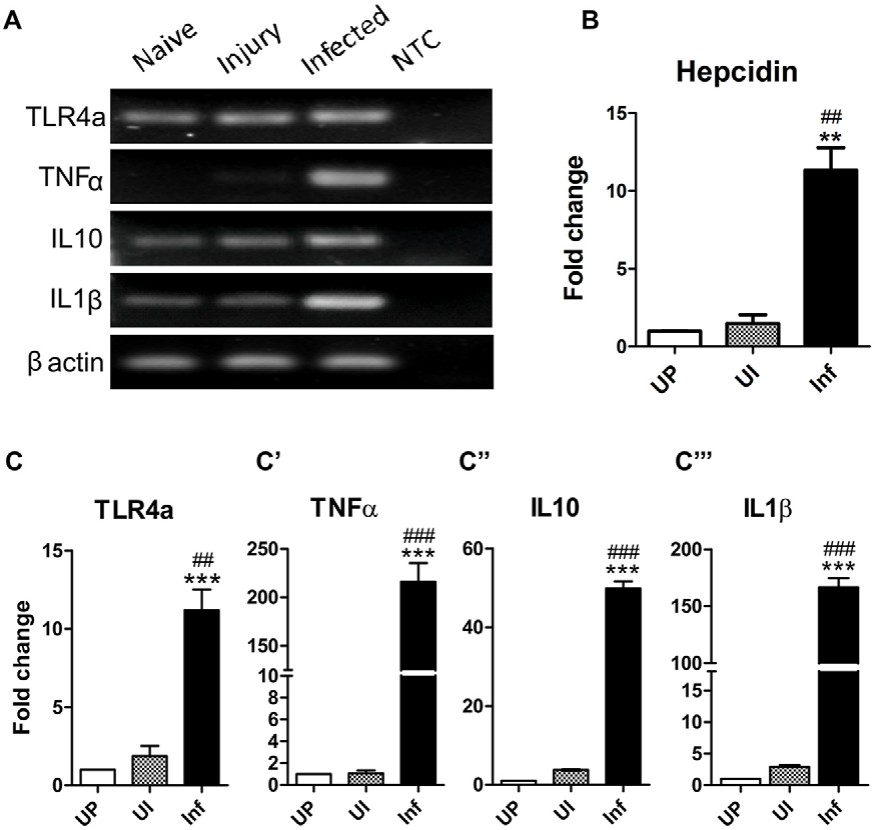
**Vertebrate skeletal muscles are immune responsive.** (A,C-C‴) Adult zebrafish myotomes induce cytokine expression upon infection. (A) Agarose gel images of expression level of cytokine genes assessed by RT-PCR. qRT-PCR analysis was performed to estimate the induction in gene expression of the zebrafish cytokine: TLR4a (C), TNFα (C′), IL10α (C″) and IL1β (C‴). (B) Induction of *Hepcidin* expression, a vertebrate AMP, upon infection with *Salmonella*. NTC, no template control; UP, unpricked; UI, uninfected with sterile injury; Inf, infected. The values represented are mean±s.e.m. of three independent replicate experiments. *, significant difference in expression of infected versus unpricked; #, infected versus uninfected. **/##, *P*<0.001; ***/###, *P*<0.0001.

Trunk muscles of infected zebrafish showed massive inductions in expression of several cytokines (Fig. 5A). Substantial inductions (∼200-fold) were evident in the expression of pro-inflammatory cytokines IL1β (Fig. 5C‴) and TNFα (Fig. 5C′), indicating an acute phase inflammatory response. Interestingly, the anti-inflammatory cytokine IL10 was also significantly induced (Fig. 5C″), albeit to a lesser extent than the pro-inflammatory cytokines. Expression of TLR4a, a Toll-like receptor 4 paralog, was also induced upon infection (Fig. 5C). Unlike *Drosophila*, where injury alone can lead to induction in AMP expression, sterile injury in zebrafish did not induce cytokines [see unpricked control (UP) versus uninfected with sterile injury (UI) in Fig. 5].

Increased expression of Hepcidin (a vertebrate AMP) has been reported following lipopolysaccharide challenge in mice (Pigeon et al., 2001) and bacterial challenge in fishes (Shike et al., 2002; Douglas et al., 2003). Trunk muscles of zebrafish also showed significant induction of expression of *Hepcidin* after bacterial challenge (Fig. 5B). This induction was specific to infection as a sterile injury did not show any change in expression.

## DISCUSSION

The results reported in this study establish the IFMs of *Drosophila* as an immune responsive tissue. IFMs produce AMPs through canonical Imd and Toll pathways in response to bacterial infections. Mutant flies with defective IFMs produce suboptimal AMPs during infection and hence are more susceptible to bacterial infections. Further, using a zebrafish infection model we establish that the immune responsiveness of skeletal muscles is evolutionarily conserved.

The susceptibility of *Drosophila* mutants to infection mirrors the degree of overall defect in their IFMs. The overall physiology of IFMs is important for an optimal immune response. This can be elaborated by two results; firstly, *wupA^hdp−3^*, an IFM-specific Troponin-I-null mutant (Nongthomba et al., 2004), displayed the highest susceptibility to infection of all the mutants examined. Another Troponin-I mutant, *wupA^hdp−2^*, had higher survival rates following infection than *wupA^hdp−3^*. This was surprising as *wupA^hdp−2^* has a missense mutation in a constitutive exon of the Troponin*-*I gene (*wupA*), resulting in the abnormal function of most muscles including the heart (Nongthomba et al., 2003; Wolf et al., 2006). Yet, *wupA^hdp−3^* mutants, with only the flight and the jump muscles affected, are more susceptible to infection. This probably results from more severely affected IFMs (that are almost absent) in *wupA^hdp−3^* compared with those in *wupA^hdp−2^* mutants , indicating that IFM, compared with other muscles in the fly body, is important for survival upon infection.

Secondly, *Act88F^KM88^* flies, which lack Actin in the IFMs (Okamoto et al., 1986), showed below-average survival upon infection. If it were the IFM mass alone that governed an efficient IFM-mediated immune response, then *Act88F^KM88^*, with the highest IFM mass, should have been more stress-resistant than the wild-type flies. If it were only IFM function, then the lowest survival rates would have been expected from *Act88F^KM88^* flies, which lack the entire thin filament structure and have disorganized thick filaments (Beall et al., 1989). An average response from *Act88F^KM88^* flies and the poorest response from *hdp^3^* flies to bacterial infection confirm that both overall bulk and physiology of the IFMs are important for survival of flies in response to infection.

Our data support an effect of muscle defects on immune function, but indirect effects of these mutations on other physiological functions, and thereby a reduced immune response, might exist. Some facts support the former. Though all mutants used in the study were flightless, flighlessness alone (flies with clipped wings and intact IFMs) does not lead to an increase in susceptibility to infection (Fig. S6). Apart from flight, motor activity of IFM mutants (walking, climbing and food uptake) is comparable to wild type (data not shown). Naïve mutant flies do not have activated stress response pathways (Fig. S3B), suggesting that they are not stressed. A reduced ability to survive infections could also be an effect of the genetic backgrounds of the fly strains. We attempted to disprove this hypothesis by utilizing mutant fly lines from different genetic backgrounds; all yielded similar results. Thus, a deficiency of IFM-mediated immune response in mutant flies might be responsible for the reduction in surviving infections.

Infections lead to release of reactive oxygen species (ROS) early on (reviewed in Rada and Leto, 2008; Dickinson and Chang, 2011). Being highly diffusible, these reactive species can act as messengers and spread the alarm to distant cells (Dickinson and Chang, 2011). Muscles have a high mitochondrial load and thus produce large amounts of ROS themselves; levels of ROS sensors and quenchers are also substantially higher in muscles (Freyssenet, 2007; Barbieri and Sestili, 2012). Moreover, muscles are more sensitive to ROS than other tissues (Godenschwege et al., 2009). This raises the possibility of muscles utilizing ROS (generated during pathogen invasion) as indicators of stress, along with other pathogen associated molecular patterns as has been reported for fat bodies during gut infections (Wu et al., 2012). Possessing such sensitive machinery makes muscles extremely perceptive to infections, therefore making it an ideal tissue to evoke immune response.

Vertebrate homologs of the *Drosophila* Imd and Toll signaling pathways are functionally active in zebrafish skeletal muscles. After 6 h of bacterial infection, zebrafish muscles show high induction (∼200-fold) of pro-inflammatory cytokines IL1β and TNFα. A similar observation in zebrafish was reported earlier (Lin et al., 2009), but the role of muscles in immune response was not suggested. It is possible that the cytokine induction we found is from muscle-resident macrophages. Murine myoblast cell lines and skeletal muscles have been reported to produce pro-inflammatory cytokines when stimulated with Gram-negative bacterial lipopolysaccharide (Frost et al., 2002). Thus, the increase in cytokine expression, at least in part, must be through muscles. Along with pro-inflammatory cytokines, zebrafish muscles also induced expression of the anti-inflammatory cytokine IL10, though to a lesser extent. The production of anti-inflammatory cytokines clearly shows that zebrafish muscles also retain the ability to inhibit an immune response when not required.

Mouse skeletal muscles produce β-defensin-6, an AMP (Yamaguchi et al., 2001). Hepcidin is another AMP that is conserved among vertebrates (Shike et al., 2004). Although expression of *Hepcidin* is highest in the liver, *Hepcidin* mRNA has also been detected in other organs such as spleen, heart and stomach (Douglas et al., 2003; Ilyin et al., 2003; Shike et al., 2004). In this study, we not only show that *Hepcidin* is expressed in zebrafish trunk muscles, but also that its expression increases upon infection. Pathways of Hepcidin induction are presently not known; nonetheless, our result reiterates that skeletal muscles can sense and effectively respond to bacterial infection.

Unlike other immune organs (liver, thymus, neurons etc.), muscles possess the unique characteristic of muscular hypertrophy (the ability to build muscles). Exercise (strength and anaerobic training) leads to both sarcoplasmic as well as myofibrillar hypertrophy, thereby increasing muscle mass and strength (Rasch and Morehouse, 1957; Schoenfeld et al., 2013; Rai and Nongthomba, 2013). Moreover, studies in rodents show that a bout of vigorous physical activity before bacterial inoculation dramatically increases their resistance to infection (Reis Gonçalves et al., 2012; Wada et al., 1990; Cannon and Kluger, 1984). Moderate physical activity enhances natural killer cell activity and immune function in general (Pedersen and Ullum, 1994; Nielsen, 2013), but whether active hypertrophic muscles are more efficient in the immune response they mount is an unanswered question. If so, exercise might boost an individual's immunity and/or fitness indirectly by affecting overall physiology as well as directly by having more ammunition against infections and stressors.

## MATERIALS AND METHODS

### Fly strains

*Canton-S* was used as the wild-type strain. The IFM mutants used in the study were *up^1^* (Nongthomba et al., 2007), *up^101^* (Nongthomba et al., 2003), *wupA^hdp−2^* (Nongthomba et al., 2003), *wupA^hdp−3^* (Nongthomba et al., 2004), *Tm2^3^* (Mogami and Hotta, 1981), *Act88F^KM88^* (Nongthomba et al., 2001)*. UH3-Gal4* (Singh et al., 2014), UAS-*Rel^RNAi^* (BS#33661), UAS-*Dif^RNAi^* (BS#30513) and *Diptericin*-LacZ (BS#55707) were procured from the Bloomington Drosophila Stock Center, Indiana. *Drosomycin*-GFP was a gift from Prof. Pradip Sinha (Indian Institute of Technology, Kanpur, India) *Drosocin*-GFP and *Cecropin*-LacZ were gifts from Dr M. Tapadia (Banaras Hindu University, Varanasi, India). Unless mentioned otherwise sample populations represented both sexes in equal proportion.

### Microbial stocks

*Salmonella enterica* subsp. *enterica*, serovar Typhimurium (strain 12023) was a gift from Prof. D. Chakravortty (Indian Institute of Science, Bangalore, India). *Escherichia coli* was a gift from Prof. S. Mahadevan (Indian Institute of Science, Bangalore, India). *Enterobacter cloacae* (MTCC #7097), *Bacillus subtilis* (MTCC #441) and *Staphylococcus aureus* (MTCC #3160) were procured from the Microbial Type Culture Collection and Gene Bank (MTCC; Imtech, Chandigarh, India).

### Infection studies

Infections were performed using the septic injury method (Apidianakis and Rahme, 2009). *Salmonella* was used for septic Gram-negative bacterial infections. Groups of at least 100 adults, aged one to two days, were randomized in two equal sets for the uninfected and infected groups (each containing equal number of males and females). Flies were pricked in the thorax using a sharpened tungsten needle dipped in the concentrated bacterial suspension (∼10^12^ colony forming units) for infection or sterile water for controls. Flies were maintained at 25°C and transferred to a fresh vial every three days. Dead flies were counted every 24 h until all the infected flies were dead.

For studies with Gram-positive infection an overnight culture of *Bacillus subtilis* or *Staphylococcus aureus* was used. The rest of the protocol was similar to the Gram-negative infection test except that dead flies were counted every hour from eight hours post-infection.

Kanamycin-resistant *Escherichia coli* was used for infections via feeding. Bacteria cultured overnight were diluted 1:2 with 5% sucrose-kanamycin solution. The diluted culture was inoculated onto LB-agar vials and incubated at 37°C for 12 h. Control vials were inoculated with 5% sucrose-kanamycin and incubated as for experimental vials. One- to two-day-old flies were introduced to these vials. Flies were transferred to fresh vials with a 12-h bacterial lawn every eight hours. Dead flies were counted every two hours until all the infected flies were dead.

Groups of five wild-type, germ-free fishes [five months old, treated with Chloramphenicol (40 mg/l for 24 h; HiMedia, Bangalore, India)] were anesthetized with Tricaine (1.68 mg/ml; Sigma-Aldrich, Bangalore, India) and infected by pricking the trunk with a sharp tungsten needle dipped in concentrated culture (∼10^12^ colony forming units) of *Salmonella*. Fish were let to recover from anesthetization and then assessed for GFP expression and survival for the next 24 h.

### Imaging

*Drosophila* hemithoraces were fixed in 4% paraformaldehyde (Sigma-Aldrich) for 30 min. The tissues were then either: (a) stained with Mayer's Hematoxylin (that stains nuclei blue-black) for 15 min, de-stained in 0.3% acid-alcohol for two minutes, serially dehydrated in 50%, 70%, 90% and 100% alcohol for one hour each and finally cleared in methyl salicylate overnight; (b) incubated in LacZ staining solution (150 mM NaCl, 1 mM MgCl_2_, 3 mM K_3_[Fe(CN)_6_], 3 mM K_4_[Fe(CN)_6_], 0.3% Triton X-100 plus 0.25% X-gal) for 10 h at 37°C; or (c) washed with phosphate-buffered saline and directly visualized after mounting. Mounting was done using DPX Mountant (Qualigens) for bright-field imaging and in Vectashield (Vector Laboratories) for fluorescence imaging. Bright-field imaging was done using an Olympus SZX12 microscope (DSS Imagetech, Bangalore, India) and the images were photographed using an Olympus C-5060 camera (DSS Imagetech). Fluorescence imaging was done using Olympus IX81 fluorescence microscope.

### qRT-PCR

Total RNA was extracted from the tissue of interest, either whole flies or muscles (according to experimental requirement), using TRIzol reagent (Sigma-Aldrich) by following manufacturer's instructions. Complementary DNA (cDNA) was prepared by using a first strand cDNA synthesis kit (RevertAid First Strand cDNA Synthesis Kit, Thermo-Scientific from Microphil, Bangalore, India) with 2 µg of RNA. The primers were used for qRT-PCRs for *Drosophila* and zebrafish genes are detailed in Table S1. qRT-PCR was run using Dynamo SYBR Green qPCR kit (Thermo-Scientific from Microphil) in a StepOnePlus™ Real Time PCR system (ABI). Experiments were repeated three times with independent biological repeats. Results were normalized to co-amplified house-keeping genes (*rp49* for *Drosophila* and *β-actin* for zebrafish). Alterations in gene expression were calculated on the basis of the relative comparative threshold cycle (Ct) value, using the ΔΔCt method. Two-tailed Mann–Whitney tests were performed for estimating statistical significance.
